# SoyDB: a knowledge database of soybean transcription factors

**DOI:** 10.1186/1471-2229-10-14

**Published:** 2010-01-18

**Authors:** Zheng Wang, Marc Libault, Trupti Joshi, Babu Valliyodan, Henry T Nguyen, Dong Xu, Gary Stacey, Jianlin Cheng

**Affiliations:** 1 Computer Science Department, University of Missouri, Columbia, MO 65211, USA; 2 Christopher S Bond Life Sciences Center, University of Missouri, Columbia, MO 65211, USA; 3 Division of Plant Sciences, National Center for Soybean Biotechnology, Christopher S Bond Life Sciences Center, University of Missouri, Columbia, MO 65211, USA; 4 Informatics Institute, University of Missouri, Columbia, MO 65211, USA

## Abstract

**Background:**

Transcription factors play the crucial rule of regulating gene expression and influence almost all biological processes. Systematically identifying and annotating transcription factors can greatly aid further understanding their functions and mechanisms. In this article, we present SoyDB, a user friendly database containing comprehensive knowledge of soybean transcription factors.

**Description:**

The soybean genome was recently sequenced by the Department of Energy-Joint Genome Institute (DOE-JGI) and is publicly available. Mining of this sequence identified 5,671 soybean genes as putative transcription factors. These genes were comprehensively annotated as an aid to the soybean research community. We developed SoyDB - a knowledge database for all the transcription factors in the soybean genome. The database contains protein sequences, predicted tertiary structures, putative DNA binding sites, domains, homologous templates in the Protein Data Bank (PDB), protein family classifications, multiple sequence alignments, consensus protein sequence motifs, web logo of each family, and web links to the soybean transcription factor database PlantTFDB, known EST sequences, and other general protein databases including Swiss-Prot, Gene Ontology, KEGG, EMBL, TAIR, InterPro, SMART, PROSITE, NCBI, and Pfam. The database can be accessed via an interactive and convenient web server, which supports full-text search, PSI-BLAST sequence search, database browsing by protein family, and automatic classification of a new protein sequence into one of 64 annotated transcription factor families by hidden Markov models.

**Conclusions:**

A comprehensive soybean transcription factor database was constructed and made publicly accessible at http://casp.rnet.missouri.edu/soydb/.

## Background

Soybean is a great source of protein, as it contains significant amounts of all the essential amino acids, including some that cannot be synthesized by the human body [1]. Soybean has been used as a food and a drug component in China for thousands of years [2] and over the past 60 years has become a leading crop in many nations around the world [3]. Because of its high value in the agricultural and food industry, soybean has received greater and greater research attention, both to improve soybean agronomic performances and as a model for basic biological studies. In early 2008, the Department of Energy-Joint Genome Institute (DOE-JGI) finished sequencing the soybean genome using a whole-genome shotgun approach [4], which makes soybean the most complex plant so far ever sequenced [5]. The homology-based gene prediction and annotation produced putative protein sequences [4,5], which makes it feasible to identify and annotate soybean transcription factors.

Transcription factors (TF) are proteins that bind to DNA sequences (*i.e.*, promoters) and regulate gene expression by one or more DNA binding domains. Virtually all biological processes are directly regulated or influenced by transcription factors [6]. For example, the transcription process in eukaryotes would not occur in the absence of a specific class of transcription factors named "general transcription factors" [7,8]. Studies have shown that transcription factors are closely involved in the process of cell development, such as cellular division, migration, and differentiation [9]. Transcription factors of *Arabidopsis thaliana *have been well studied since its genome has been fully sequenced as a model specie [6,10-14]. This makes it possible to identify and study transcription factors of other newly sequenced species, such as soybean, by homology searching and comparative analysis.

Several databases for soybean genome analysis have been built and made publicly available, such as SoyGD [15], SoyBase [16], and SoyXpress [17]. These databases contain a variety of information, such as soybean genome sequences, bacterial artificial chromosome (BAC), expressed sequence tags (EST), and some useful tools including genome browsers, BLAST searching, and pathway searching. However, these databases only contain general annotations for the soybean genome, instead of knowledge specifically targeting the transcription factors. For example, none of them systematically organizes transcription factors into families or clearly points out the DNA binding domains. PlantTFDB [18] and DBD [19] are two existent transcription factor databases, which contain knowledge about transcription factors from multiple species. For each soybean transcription factor, PlantTFDB contains information including protein sequence, Gene Ontology annotation [20], putative binding domains found by InterProScan [21], and cross-links to external databases, including EMBL [22], UniProt [23], RefSeq [24], and TRANSFAC [25]. DBD contains the amino acid sequence of each transcription factor and external links to Ensembl [26], Pfam [27], and SUPERFAMILY [28]. Compared to PlantTFDB, DBD has less external database links, but DBD claims to contain the transcription factors of 927 completely sequenced genomes whereas PlantTFDB covers 22 species. The knowledge in these two databases is very useful; however, they were built based on a relatively older version of soybean sequence data and their annotations are still incomplete. The most important component they lack is the three dimensional structure for each transcription factor, because the visualization of the transcription factor, especially binding sites, can help further understanding the mechanism and functions of transcription factors, which is indispensible to structural genomics [29,30]. Furthermore, with the complete genome sequences of more and more species available, a computer system is needed that can automatically generate a knowledge database and publish it with a user-friendly interface.

To fill the gap, we developed SoyDB - a comprehensive and integrated database for soybean transcription factors. This database not only contains most of the content and features already existed in PlantTFDB and DBD, but also extends them by containing more comprehensive knowledge and links to more versatile external datasets. The annotations in SoyDB include predicted tertiary structures, protein domains, multiple sequence alignments, DNA binding sites, and web logos and consensus sequences for each family. The SoyDB database also contains links to the homologous EST sequences, and the same or homologous proteins in other databases including PlantTFDB, PDB [31], Swiss-Prot [32], TAIR [33], RefSeq [24], SMART [34], Pfam [27], KEGG [35], SPRINTS [36], EMBL [37], InterPro [38], PROSITE [39], and Gene Ontology [20].

Moreover, our system can automatically execute bioinformatics tools and generate annotations, link to other well-known protein databases, construct MySQL databases, and generate PHP scripts to build its website. This fully automated approach can be used to create a protein annotation database and website for any sequenced organism in the future.

## Construction and Content

### Database Overview

**Figure 1:**
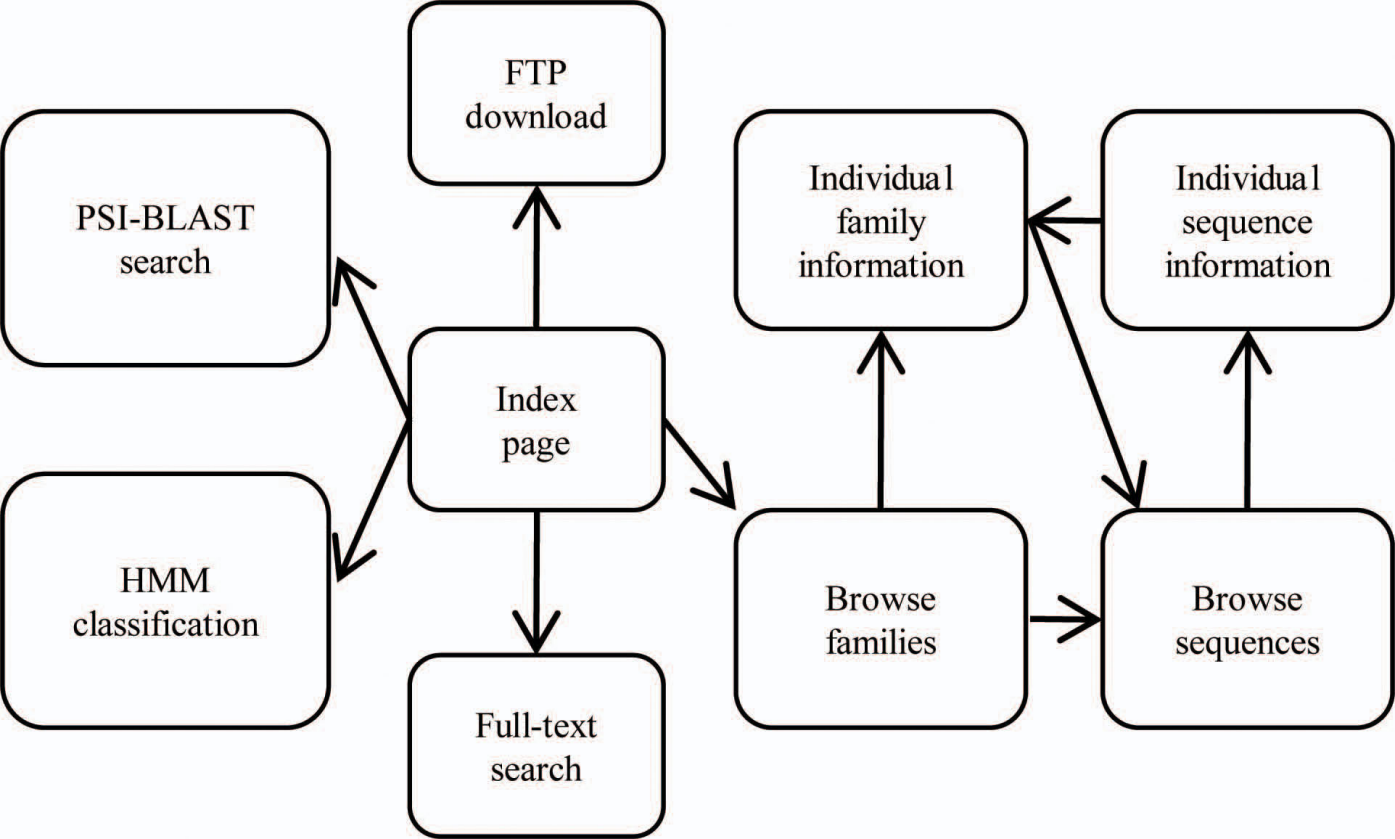
**Architecture of SoyDB website**. Main annotation components include family information page and sequence information page. Main functional components include full-text search, HMM family classification, PSI-BLAST sequential search, and FTP downloading.

SoyDB contains the annotations of 5,671 putative transcription factors. These proteins were classified into 64 families (for details see the section "Transcription Factor Family Prediction Using SAM Hidden Markov Models"). Figure 1 illustrates the architecture of the SoyDB website. Users can access the main components from the home page: full-text search, PSI-BLAST sequence search, family classification by hidden Markov model, family browsing, protein browsing, family information, protein information, and FTP site.

### Data Source

The soybean genome sequences and gene model predictions used in this study were acquired from the publicly available database Phytozome [5]. These sequences were generated by the preliminary GenomeScan [40], FgenesH [41], and PASA [42] gene annotations based on the Gm1.01 version of soybean assembly data [4].

### Transcription Factor Identification

We used the standalone versions of InterProScan [21] to search all the soybean protein sequences against 11 databases integrated in InerPro [38]. These databases and their corresponding scanning methods include: PROSITE (*pfscan*) [39], PRINTS (*FingerPRINTScan*) [43], Pfam (*HMMPfam*) [27], ProDom (*ProDomBlast3i*) [44], SMART (*HMMSmart*) [34], TIGRFAMs (*HMMTigr*) [45], PIR SuperFamily (*HMMPIR*) [46], SUPERFAMILY (*superfamily*) [47], Gene3D (*gene3d*) [48], PANTHER (*HMMPanther*) [49], and HAMAP (*pfscan*) [50]. InterProScan systematically searches each of these databases using their corresponding scanning methods to find domains. The proteins predicted to contain TF related domain(s) were considered as putative transcription factors. Using the Plant Transcription Factor Database (PlnTFDB) [51] and the classification of *Medicago truncatula *TF genes [52] as references, we manually curated the list of putative transcription factors and eliminated any mistakenly identified sequences. In this way, we identified 5,671 putative TF sequences.

### Transcription Factor Family Prediction Using SAM Hidden Markov Models

The transcription factors of *Arabidopsis thaliana *have been well studied and classified into 64 families [33]. This provides a model for us to classify soybean transcription factors. We used MUSCLE [53] to generate a multiple sequence alignment for each *Arabidopsis thaliana *TF family. The multiple sequence alignment was then input into SAM 3.5 [41] to build a hidden Markov model (HMM) for each family. Every soybean TF sequence was aligned with each of the 64 HMMs, which outputs an e-value. This e-value can be considered as a fitness score between a TF sequence and a hidden Markov model: lower e-value indicates better fitness. Finally, a transcription factor was classified into the family whose HMM yields the lowest e-value.

### Annotations Using Bioinformatics Tools

**Figure 2:**
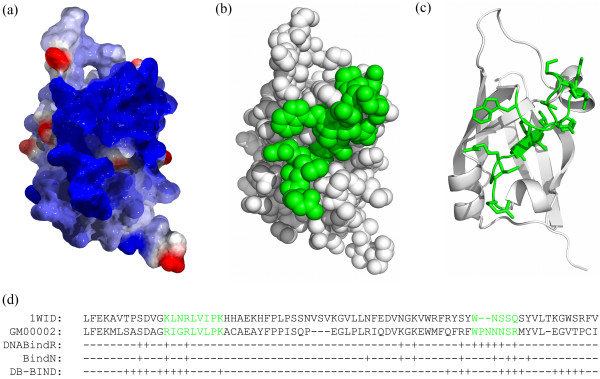
**The predicted structure of a transcription factor in SoyDB**. The electrostatic polarization (a) (blue, positive; red, negative), sphere (b) and ribbon (c) visualizations of MULTICOM predicted structure for GM00002. (d) a segment of the pair-wise alignment between 1WID (PDB template of GM00002) and GM00002, and, below, the DNA-binding site predictions from three independent tools: DNABindR [70], BindN [71], and DP-Bind [72] ("+" indicates predicted DNA-binding positions, "-" indicates gap or no prediction). The green regions in the sequence of 1WID are the DNA-binding regions identified by experimental methods [73]. The green regions in GM00002 sequence are the two DNA-binding regions derived from the alignment with 1WID. The predicted DNA-binding regions in GM00002 are illustrated in green in (b) and (c). (c) the side chains of the predicted binding regions. (a), (d), and (c) are in the same orientation. The electrostatic polarization (a) was computed and mapped to protein surface by Swiss-PDB viewer (deep view) [74], and the structures in sphere (b) and ribbon styles (c) were made with PyMol [75].

The standalone versions of several bioinformatics tools were locally installed and executed to generate annotations for soybean transcription factors. An accurate protein structure prediction tool MULTICOM [54] was used to predict the tertiary structure of each transcription factor when homologous template proteins could be found in PDB. According to the official evaluations of the 8^th ^community-wide Critical Assessment of Techniques for Protein Structure Prediction (CASP8) [55], MULTICOM was able to predict high-accuracy tertiary structures with an average GDT-TS score [56] 0.87 if suitable templates can be found. GDT-TS score ranges from 0 to 1 measuring the similarities between the predicted and experimental structures, whereas 1 indicates completely the same and 0 completely different. Figure 2 illustrates the predicted tertiary structure of a transcription factor in SoyDB with ID GM00002, and the electrostatic polarization of the predicted structure. The blue area in the electrostatic polarization shows residues positively charged, which is found to be highly identical to the green area in Figure 2(b), which is the putative DNA-binding sites identified by a pair-wise alignment between GM00002 and its template protein 1WID (Figure 2(d)). Since it has been studied and found that the DNA-binding area is positively charged if analyzed by electrostatic potentials [57], the highly identical area in Figure 2(a) and 2(b) strongly confirms that the predicted structure has the electrostatic properties of a transcription factor. This further confirms the qualities of MULTICOM predictions and the correctness of the predicted binding sites derived from the homology alignment. In SoyDB, a predicted tertiary structure is visualized by Jmol [58]. In order to clearly visualize the tertiary structure of the DNA-binding region, only the segments containing homologous DNA binding domains are visualized by Jmol. Users can view a TF structure from various perspectives in a three-dimensional way and perform many operations including selecting and highlighting interested regions, changing view styles and colors, and measuring atom distances and angles by right clicking on the Jmol console and selecting corresponding menus. Detailed instructions about Jmol menus can be found at Jmol website [58]. During the structure prediction process, MULTICOM generates the sequence alignments between the transcription factor and its homologous templates using PSI-BLAST. These sequence alignments can be used to predict the binding sites of a transcription factor based on the experimentally determined binding sites of its template as shown in Figure 2.

A predicted structure was parsed into domains by Protein Domain Parser (PDP) [59]. Since some transcription factors did not have homologous templates found in PDB, DOMAC [60], an accurate *ab initio *domain prediction tool, was also used to predict domains for each transcription factor.

The protein sequences in the same family were aligned by MUSCLE [53] and visualized by WebLogo [61]. A consensus sequence was derived from the multiple sequence alignment by selecting the most frequently appeared amino acid at each position. The multiple sequence alignments were also used to identify the conserved signatures (likely the DNA binding domains) for each family.

All of the bioinformatics tools incorporated to construct SoyDB can be used to automatically annotate other species in the future.

### Links to External Databases and Datasets

In order to annotate the functions of soybean transcription factors, each TF protein sequence was searched against the soybean TF database PlantTFDB, NCBI known EST sequences, and other general protein databases by PSI-BLAST or TBLASTN. The external protein databases include Swiss-Prot [32], TAIR [33], RefSeq [24], SMART [34], Pfam [27], KEGG [35], SPRINTS [36], EMBL [37], InterPro [38], PROSITE [39], and Gene Ontology [20]. Web links to these databases were created when the same transcription factor or its homologous proteins were found in them; and for each database or EST dataset only the PSI-BLAST or PBLASTN hit with the smallest e-values was listed in SoyDB. To search the known EST sequences, PSI-BLAST was first used to build a position-specific score matrix for each transcription factor. TBLASTN was then used to search each protein sequence against three known EST datasets: EST human, EST mouse, and EST others. GenBank [62] web page of each EST hit was linked to SoyDB website. The gene expression of a subset of TF genes (about 1,000 TF genes) was recently published [63]. Transcription profile of all soybean TF genes in various conditions is under investigation.

These external links greatly expand the annotation scope of SoyDB providing related knowledge from various perspectives. SoyDB provides a systematic view of a transcription factor -- from the features of the protein itself, to the biological pathway it locates in. The links to the external databases and datasets can be updated by a re-run of PSIBLAST and TBLASTN. Currently, these links are scheduled to be updated once every six months. This time interval can be changed if necessary.

### Database and Website Implementation

The programs used to automatically annotate proteins were written in PERL. The relational database was built on MySQL with database schemas automatically generated by programs written in PERL. The website was implemented in PHP. The database and web site were automatically constructed by computer programs with little human intervention.

## Utility and Discussion

### Protein Information

**Figure 3:**
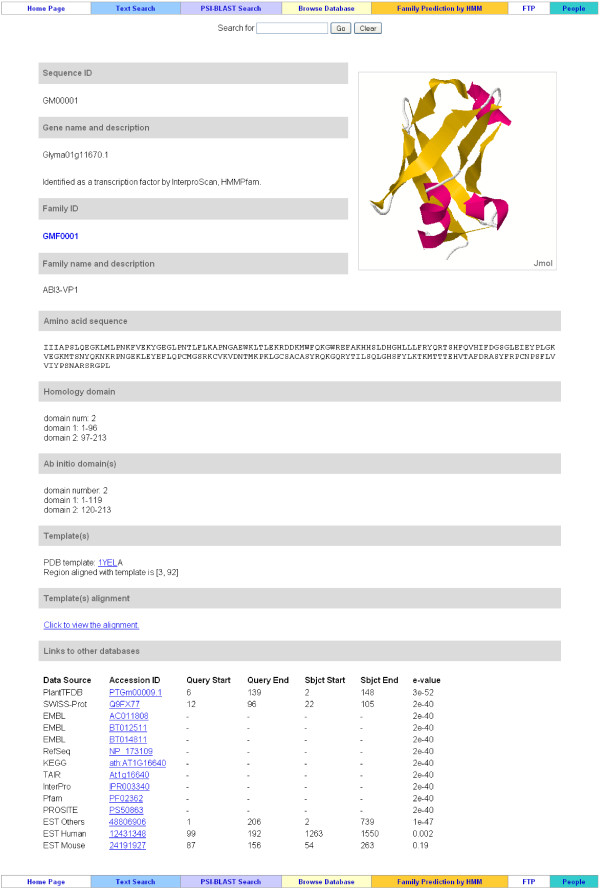
**Information page for a transcription factor**. This example web page shows the knowledge for each transcription factor, which includes amino acid sequence, predicted tertiary structure, domain(s) found by homologous search and *ab initio *prediction, PDB template and alignment, and links to other protein databases and EST datasets.

This component contains the complete annotations for each transcription factor, including protein ID, protein name and description, tools used for TF identification, family ID, family name and description, amino acid sequence, homology domain prediction, *ab initio *domain prediction, PDB homologous templates, and predicted tertiary structure. This component can be reached by clicking the sequence ID, such as GM00001, or the Phytozome protein name, such as Glyma01g11670.1, at the "Protein Browsing" webpage (for details see the following "Protein Browsing" section). Figure 3 illustrates the protein information page. The sequence ID and family ID, such as GM00001 and GMF0001, are internal indices used by the SoyDB, and the sequence name is the standard soybean TF name used by the soybean genome database Phytozome [5]. We noticed the trend of unifying annotation formats within the soybean community. Therefore, the commonly used TF ID format, such as PTGm00009.1, is also compatible in SoyDB. Details are described in the "Full-Text Search" section below.

### Family Information

**Figure 4:**
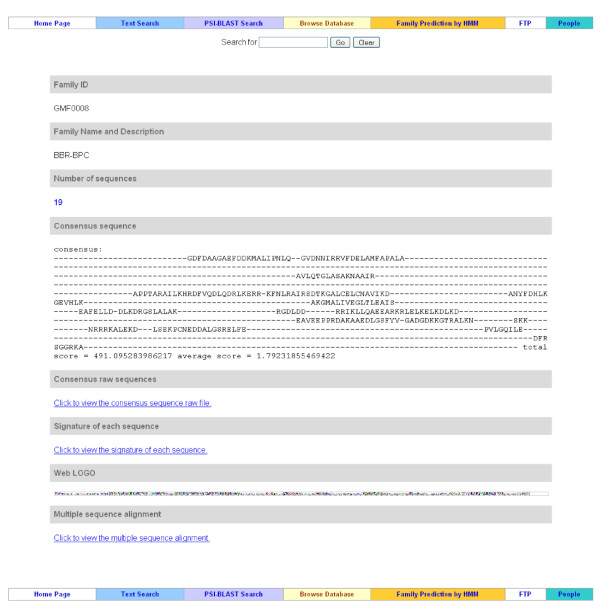
**Family information page**. This example web page shows the knowledge for each TF family, which includes number of sequences in the family, consensus sequence of the family, signature of sequences in the family, web logo, and multiple sequence alignment of the sequences in the family.

This component contains the complete annotations for each TF family, including family ID, family name and description, number of sequences within the family, consensus sequence, consensus signatures (likely the DNA binding regions), web logo of the signature profile, and multiple sequence alignment of the protein sequences within the family. Figure 4 demonstrates a family information web page. This component can be reached from the "Family Browsing" web page.

### Protein Browsing

**Figure 5:**
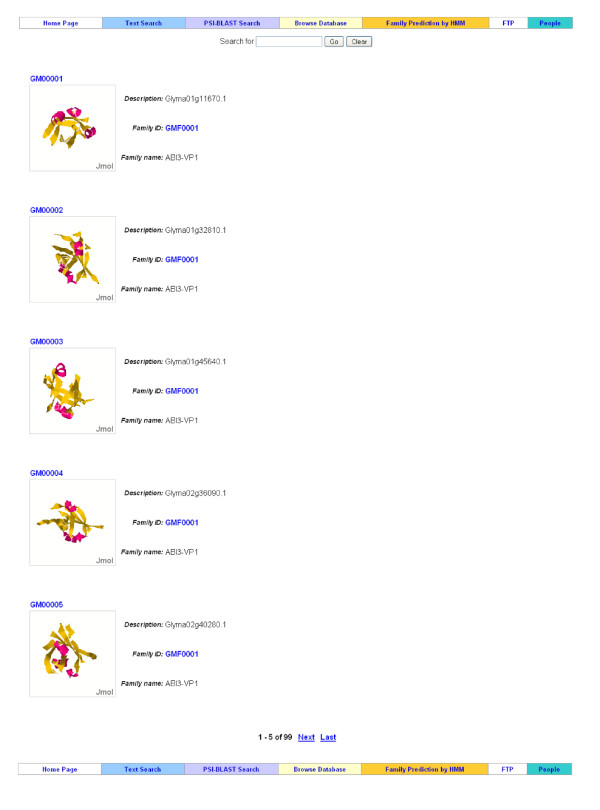
**Transcription factor browsing page**. This page lists the transcription factors in a TF family. The tertiary structure of each sequence is displayed in an interactive way, *i.e.*, users can zoom in/out and rotate the structure. Users can further view sequence annotations by clicking the TF IDs, and view family annotations by clicking family names.

The transcription factors within a family are listed in the order of sequence IDs. The list contains the thumbnail of tertiary structure, protein ID and name, family ID, and family name of each transcription factor (Figure 5). Users can further view the complete annotations by clicking its sequence ID or the Phytozome protein name. This component can be reached by clicking the number of sequences in the "Family Browsing" or the "Family Information" web page.

### Family Browsing

A user can browse SoyDB from TF family perspective. The 64 TF families are listed in the order of family IDs. The family ID, family name, and the number of transcription factors within each family are listed. By clicking the family ID or name, users can view the complete annotations for a family, or further browse the sequences within a family by clicking the number of sequences. This component can be reached by clicking "Browse Database" in both the top and bottom menu bars from any SoyDB web pages. Additional file 1 (**Figure S1**) illustrates the web page showing a TF family list.

### Full Text Search

This component allows users to search the entire SoyDB database by a query text, such as protein name or family name. Given input keywords, SoyDB searches all the fields in the database and returns matched transcription factors with links to their annotations. Users can also search SoyDB by the TF IDs used in PlantTFDB. The search component will return the homologous soybean TFs found in SoyDB.

### PSI-BLAST Sequence Search

This component allows users to search a query sequence against every TF sequence stored in SoyDB. Users can submit a query sequence and adjust PSI-BLAST parameters from a web page as shown in Additional file 2 (**Figure S2**). After a PSI-BLAST search is performed, the significant hits, with links to their annotation web pages, are ranked based on the e-values generated by PSI-BLAST. Additional file 3 (**Figure S3**) illustrates a PSI-BLAST result web page.

### Family Classification by Hidden Markov Model

This component classifies a query protein sequence into one of the 64 TF families. Additional file 4 (**Figure S4**) illustrates the web page for family classification. A submitted query sequence is aligned with each of the 64 hidden Markov models built based on the 64 *Arabidopsis thaliana *TF families. The query sequence is classified into a family whose hidden Markov model outputs the lowest e-value (correspondingly the highest alignment score or fitness score). More details about family classification can be found at the "Transcription Factor Family Prediction Using SAM Hidden Markov Models" section under "Construction and Content".

### FTP Site

All of the information in SoyDB is available for users to download from an FTP site. For example, users can download all of the TF protein sequences in the FASTA format and the multiple sequence alignments for each family in plain text. This makes it possible for other websites to link to SoyDB by performing PSI-BLAST searches on SoyDB sequences, similarly as SoyDB links with other external databases.

### Comparisons and Overlapping between SoyDB and PlantTFDB

In total, SoyDB has 5,671 transcription factors - 4,306 of them (75.9%) have hits found in PlantTFDB identified by PSI-BLAST with an e-value threshold 10^-3^. PlantTFDB has 1,891 soybean transcription factors (based on the FASTA file downloaded from PlantTFDB FTP site), and 1,805 of them (95.5%) have hits found from SoyDB based on a PSI-BLAST search with an e-value threshold 10^-3^.

### Comparisons of Soybean Transcription Factor Family Distributions with Other Plants

The collection and analyses in SoyDB allows us to perform comparisons of TF family distributions across the plant kingdom. The large number of soybean TF genes (5,671) described in this study is likely due to the two soybean whole genome duplication events; one estimated to have occurred 40-50 million years ago (mya) and the most recent one approximately 10-15 million years ago [64,65]. By comparing the total number of genes in different organisms, it was found that the increase of plant gene number is related to multicellularity and ploidy. For example, compared to the unicellular eukaryote *Chlamydomonas reinhardtii *where 15,143 genes were predicted [66], larger numbers of protein-encoding genes were reported in multicellular plant organisms, *e.g.*, *Physcomitrella patens *(35,938; [67]), *Arabidopsis thaliana *(32,944; TAIR [33]), and the tetraploid *Glycine max *(66,153, Phytozome). We hypothesize that TF gene number also follows the same trend as land plants, which have a larger number of TF genes compared to algae. To perform comparisons of plant TF genes and their distributions across TF gene families, we mined the last updated DBD database [19] for 11 plant species (*C. reinhardtii, P. patens, Oryza sativa, Zea mays, Sorghum bicolor, Lotus japonicum, Medicago truncatula, A. thaliana, Vinis vinifera, Ricinus communis*, and *Populus trichocarpa*). These species were then compared with the soybean TF genes stored in our SoyDB database.

**Figure 6:**
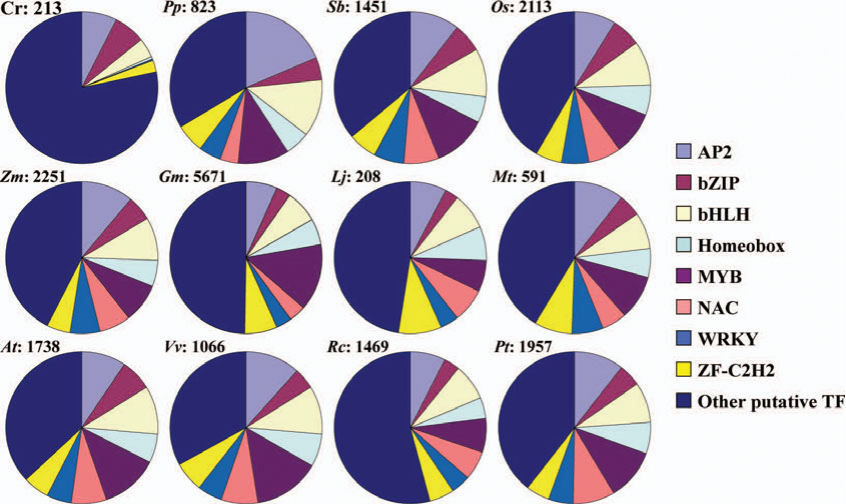
**Distributions of transcription factor families across major plant species**. Phytozome and DBD databases were mined to identify transcription factor genes in soybean (Gm: Glycine max) and in the 11 remaining plant species, respectively (Cr: Chlamydomonas reinhardtii; Pp: Physcomitrella patens; Sb: Sorghum bicolor; Os: Oryza sativa; Zm: Zea mays; Lj: Lotus japonicus; Mt: Medicago truncatula; At: Arabidopsis thaliana; Vv: Vinis vinifera; Rc: Ricinus communis; Pt: Populus trichocarpa). After being classified based on their family membership, nine major TF families are represented for each plant species. Numbers next to the plant name abbreviation are the total number of TF genes available in DBD. Details are available in Table 1.

Our analysis showed that the unicellular *C. reinhardtii *has the lowest number of TF genes compared to multicellular land plants (the exceptions are *L. japonicus *and *M. truncatula *where only a partial genome sequence is available). This trend also reflects the differences of total gene number in the organisms shown in Figure 6. For example, it is interesting to note that homeobox, MYB, NAC, and WRKY TF genes in *C. reinhardtii *lack or have very low representations compared to the 11 other plant models (Table 1). Previous studies defined a role for homeobox [68] and WRKY genes [13] in plant development. Therefore, the occurrence of these genes only in multicellular plants may reflect their special roles in development. In addition, a close relationship between TF gene number and total gene number [69] is observed when comparing the TF gene numbers of *G. max *and *A. thaliana *with their total gene numbers (*i.e.*, *G. max *encodes 66,153 protein-coding genes including 5,683 TF genes; *A. thaliana *encodes 32,944 protein-coding genes and 1,738 TF genes). Thus, the family distribution of soybean TF genes is similar to other land plant species, except for *P. patens *(*e.g.*, AP2 represents 7% of total TF genes in soybean vs. 8-12% for other land plants; bZIP: 3% vs. 3-7%; bHLH: 7% vs. 8-11%; homeobox: 6% vs. 4-7%; MYB: 14% vs. 7-14%; NAC: 4% vs. 4-9%; WRKY: 3% vs. 4-7%; ZF-C2H2: 7% vs. 5-9%) (Figure 6 and Table 1).

**Table 1:** Distributions of transcription factor families across major plant species

	At	Zm	Os	Gm	Lj	Mt	Sb	Pt	Pp	Cr	Vv	Rc
AP2	162	251	186	381	16	63	153	207	153	16	124	111
bZIP	116	119	130	176	6	27	89	90	38	14	48	50
bHLH	183	207	203	393	16	47	148	172	102	9	110	112
homeobox	105	121	132	319	15	35	81	129	44	1	74	66
MYB	212	192	193	791	14	56	165	210	89	0	151	105
NAC/NAM	132	149	146	208	15	31	111	174	32	0	81	92
WRKY	89	141	123	197	8	39	92	103	37	1	59	58
ZF-C2H2	98	114	117	395	19	49	88	101	51	5	67	78
Other TF	641	957	883	2823	99	244	524	771	277	167	352	797

Total	1738	2251	2113	5671	208	591	1451	1957	823	213	1066	1469

												

**%**	**At**	**Zm**	**Os**	**Gm**	**Lj**	**Mt**	**Sb**	**Pt**	**Pp**	**Cr**	**Vv**	**Rc**

AP2	9%	11%	9%	7%	8%	11%	11%	11%	19%	8%	12%	8%
bZIP	7%	5%	6%	3%	3%	5%	6%	5%	5%	7%	5%	3%
bHLH	11%	9%	10%	7%	8%	8%	10%	9%	12%	4%	10%	8%
homeobox	6%	5%	6%	6%	7%	6%	6%	7%	5%	0%	7%	4%
MYB	12%	9%	9%	14%	7%	9%	11%	11%	11%	0%	14%	7%
NAC/NAM	8%	7%	7%	4%	7%	5%	8%	9%	4%	0%	8%	6%
WRKY	5%	6%	6%	3%	4%	7%	6%	5%	4%	0%	6%	4%
ZF-C2H2	6%	5%	6%	7%	9%	8%	6%	5%	6%	2%	6%	5%
Other TF	37%	43%	42%	50%	48%	41%	36%	39%	34%	78%	33%	54%

Total	100%	100%	100%	100%	100%	100%	100%	100%	100%	100%	100%	100%

Collectively, these results suggest that soybean TF genes were not lost following soybean genome duplication, and may have evolved for specialized functions in plant development or response to the environment.

### Future Development Plan

In the future, we plan to link to more soybean database, such as SoyBase, and add a human expert discussion section for each transcription factor where biologists can register, log in, and make comments on any annotation items. Also, we plan to link the protein name, such as Glyma01g11670.1, listed in each protein information page to its entry in Phytozome. By doing this, SoyDB can be linked with other soybean genome annotations. Furthermore, we may identify the binding regions on the soybean DNA sequences, which can further help biologists target the regulated regions on soybean genome.

## Conclusions

SoyDB is a comprehensive database for soybean transcription factors. It integrates bioinformatics tools and various external databases to provide rich annotations, which can be browsed and retrieved through convenient web interfaces. The automated process generates annotations and creates database and website, and can be used to annotate other sequenced species.

## Availability and Requirements

SoyDB is freely available at http://casp.rnet.missouri.edu/soydb/ for academic use. Based on our test, SoyDB is fully functional with three web browsers: Mozilla Firefox, Internet Explorer, and Safari, and four operating systems: Windows XP, Windows Vista, Linux (Red Hat), and Mac OS. The only system requirement for SoyDB is that JAVA runtime environment (JRE) needs to be installed and set fully functional in order to make Jmol work.

## Authors' contributions

JC conceived the idea of automating the process of protein annotation and database construction, and coordinated the project. JC and ZW designed the database and formulated the content of the annotations. JC developed or executed the tools that generate structures, templates, domains, DNA binding sites, and multiple sequence alignments. ZW implemented the computer system that automatically gathers data, constructs database, builds web site, and links to external databases and data sources. ZW, TJ, ML and JC performed the soybean TF family classification. ZW, JC, ML, and TJ wrote the first draft of the manuscript. ML analyzed and compared the distributions of transcription factors in multiple species. TJ and ML identified the list of transcription factors. GS, DX, HN, and BV contributed some annotation ideas. JC, GS, DX, and HN provided financial support for the project. All edited and approved the manuscript.

## Supplementary Material

Additional file 1 **Figure S1 The SoyDB web page showing a list of transcription factor families**. TF families are shown with their family ID, family name, and number of sequences within the family. Click on the family ID can further view the detailed information about the family as shown in Figure 4, and click on the number of sequences can open the webpage showing all the transcription factors within the family, as shown in Figure 5. Click here for file

Additional file 2 **Figure S2 The PSI-BLAST search web page**. Users can paste or type in a query amino acid sequence and specify PSI-BLAST parameters on the web page. Click on the "Run" button will execute PSI-BLAST. Click here for file

Additional file 3 **Figure S3 The result web page of PSI-BLAST search**. PSI-BLAST result page shows the hit TF sequence ID, and the PSI-BLAST score and E-value. The hits are listed in a decreasing order of the PSI-BLAST score. Click on the sequence ID can open the web page showing detailed TF information, as shown in Figure 3. Click here for file

Additional file 4 **Figure S4 The HMM family classification web page**. Users can paste or type in a query amino acid sequence. Click on the "Predict" button will execute family classification by HMM. Click here for file
