# Supplementary material for: SoyDB: a knowledge database of soybean transcription factors
Source: BMC Plant Biol. 2010 Jan 18;10:14. doi: 10.1186/1471-2229-10-14 (PMC2826334; doi:10.1186/1471-2229-10-14)
Supplement: Additional file 1 — Figure S1 The SoyDB web page showing a list of transcription factor families. TF families are shown with their family ID, family name, and number of sequences within the family. Click on the family ID can further view the detailed information about the family as shown in Figure 4, and click on the number of sequences can open the webpage showing all the transcription factors within the family, as shown in Figure 5. [file 1471-2229-10-14-S1.PDF]

Search for [ ] Go Clear

Family ID	Family name	No. of sequences
GMF0001	ABI3-VP1	99
GMF0002	Alfin	45
GMF0003	AP2-EREBP	426
GMF0004	ARF	101
GMF0005	ARID	62
GMF0006	AS2	96
GMF0007	AUX-IAA	107
GMF0008	BBR-BPC	19
GMF0009	BES1	21
GMF0010	bHLH	559
GMF0011	bZIP	235
GMF0012	C2C2-CO-like	118
GMF0013	C2C2-DOF	102
GMF0014	C2C2-GATA	113
GMF0015	C2C2-YABBY	46
GMF0016	C2H2	392
GMF0017	C3H	197
GMF0018	CAMTA	92
GMF0019	CCAAT-DR1	1
GMF0020	CCAAT-HAP2	25

1 - 20 of 64 Next Last

Home Page	Text Search	PSI-BLAST Search	Browse Database	Family Prediction by HMM	FTP	People
