# Supplementary material for: SoyDB: a knowledge database of soybean transcription factors
Source: BMC Plant Biol. 2010 Jan 18;10:14. doi: 10.1186/1471-2229-10-14 (PMC2826334; doi:10.1186/1471-2229-10-14)
Supplement: Additional file 2 — Figure S2 The PSI-BLAST search web page. Users can paste or type in a query amino acid sequence and specify PSI-BLAST parameters on the web page. Click on the "Run" button will execute PSI-BLAST. [file 1471-2229-10-14-S2.PDF]

Use PSI-BLAST to find hits from our database

Please paste or type in the query amino acid sequence below:

```
IIIAPSLQEGKLMLPNKFVEKYGEGLPNTLFLKAPNGAEWKLTLEKRDDKMWFQKGWREFAKHHSLDHGHLL[illegible]
VEGKMTSNYQKNKRPNGEKLEYEFLQPCMGSRKCVKVDNTMKPKLGCSACASYRQKGQRYTILSQLGHSFYL[illegible]
VIYPSNARSRGPL
```

e-value: [ ] (Default value is 0.001)

iteration number: [ ] (Default value is 3)

(This process may take more than 30 seconds, depending on the number of iterations and the number of hits found from this database. Please wait after clicking the "Run" button.)

Run | clear

Home Page | Text Search | PSI-BLAST Search | Browse Database | Family Prediction by HMM | FTP | People
