# Supplementary material for: SoyDB: a knowledge database of soybean transcription factors
Source: BMC Plant Biol. 2010 Jan 18;10:14. doi: 10.1186/1471-2229-10-14 (PMC2826334; doi:10.1186/1471-2229-10-14)
Supplement: Additional file 3 — Figure S3 The result web page of PSI-BLAST search. PSI-BLAST result page shows the hit TF sequence ID, and the PSI-BLAST score and E-value. The hits are listed in a decreasing order of the PSI-BLAST score. Click on the sequence ID can open the web page showing detailed TF information, as shown in Figure 3. [file 1471-2229-10-14-S3.PDF]

Search for [ ] Go Clear

The following are the sequence(s) found:

Sequence ID	Score	E-value
GM00001	304	8e-84
GM00056	250	9e-68
GM00064	233	9e-63
GM00075	208	4e-55
GM00068	207	7e-55
GM00036	199	3e-52
GM00026	198	3e-52
GM00074	198	5e-52
GM00025	196	1e-51
GM00037	196	1e-51
GM00078	182	2e-47
GM00019	182	3e-47
GM00030	178	6e-46
GM00023	177	1e-45
GM00015	164	7e-42
GM00035	164	7e-42
GM00031	163	2e-41
GM00022	161	5e-41
GM00027	158	4e-40
GM00048	156	2e-39

1 - 20 of 53 Next Last
