# Supplementary material for: SoyDB: a knowledge database of soybean transcription factors
Source: BMC Plant Biol. 2010 Jan 18;10:14. doi: 10.1186/1471-2229-10-14 (PMC2826334; doi:10.1186/1471-2229-10-14)
Supplement: Additional file 4 — Figure S4 The HMM family classification web page. Users can paste or type in a query amino acid sequence. Click on the "Predict" button will execute family classification by HMM. [file 1471-2229-10-14-S4.PDF]

Classify a query sequence into one of 64 transcription factor families

Please paste or type in the query amino acid sequence below:

```
IIIAPSLQEGKLMLPNKFVEKYGEGLPNTLFLKAPNGAEWKLTLEKRDDKMWFQKGWREFAKHHSLDHGHLL
VEGKMTSNYQKNKRPNGEKLEYEFLQPCMGSRKCVKVDNTMKPKLGCSACASYRQKGQRYTILSQLGHSFYL
VIYPSNARSRGPL
```

(This process may take about 10 seconds, depending on the length of query sequence. Please wait after clicking the "Predict" button.)

Predict | Clear
